# Quantitative Distribution of Cerebral Venous Oxygen Saturation and Its Prognostic Value in Patients with Acute Ischemic Stroke

**DOI:** 10.3390/brainsci12081109

**Published:** 2022-08-20

**Authors:** Fengqiu Cao, Mingming Wang, Shanhua Han, Shengyu Fan, Yingwei Guo, Yingjian Yang, Yu Luo, Jia Guo, Yan Kang

**Affiliations:** 1 College of Medicine and Biological Information Engineering, Northeastern University, Shenyang 110169, China; 2 College of Health Science and Environmental Engineering, Shenzhen Technology University, Shenzhen 518118, China; 3 Department of Radiology, School of Medicine, Shanghai Fourth People’s Hospital Affiliated to Tongji University, Shanghai 200434, China; 4 Department of Psychiatry, Columbia University, New York, NY 10027, USA; 5 Engineering Research Centre of Medical Imaging and Intelligent Analysis, Ministry of Education, Shenyang 110169, China; 6 School of Applied Technology, Shenzhen University, Shenzhen 518060, China

**Keywords:** quantitative distribution, cerebral venous oxygen saturation, hypoxic regions, acute ischemic stroke, prognosis

## Abstract

This study investigated the quantitative distribution of cerebral venous oxygen saturation (SvO2) based on quantitative sensitivity mapping (QSM) and determined its prognostic value in patients with acute ischemic stroke (AIS). A retrospective study was conducted on 39 hospitalized patients. Reconstructed QSM was used to calculate the cerebral SvO2 of each region of interest (ROI) in the ischemic hemisphere. The intraclass correlation coefficient (ICC) and Bland–Altman analysis were conducted to define the best resolution of the distribution map. The correlation between the cerebral SvO2 in hypoxic regions (SvO2_ROI_ < 0.7) and clinical scores was obtained by Spearman and power analysis. The associations between cerebral SvO2 and unfavorable prognosis were analyzed using multivariate logistic regression. Excellent agreement was found between the cerebral SvO2 in hypoxic regions with a resolution of 7.18 × 7.18 × 1.6 mm^3^ and asymmetrically prominent cortical veins regions (ICC: 0.879 (admission), ICC: 0.906 (discharge)). The cerebral SvO2 was significantly negative with clinical scores (all |r| > 0.3). The cerebral SvO2 and its changes at discharge were significantly associated with an unfavorable prognosis (OR: 0.812 and 0.866). Therefore, the cerebral SvO2 in hypoxic regions measured by the quantitative distribution map can be used as an indicator for evaluating the early prognosis of AIS.

## 1. Introduction

Acute ischemic stroke (AIS) accounts for 67.3–80.5% of all strokes, with high incidence, mortality, disability, and recurrence rates [1]. Accurate diagnosis of ischemic stroke and prediction of post-treatment risk has always been the focus of imaging research, which is directly related to the choice of treatment strategies. Clinicians cannot only rely on the time window to select patients for treatment. Recent studies have shown that patients outside the time window can also benefit from active treatment [2]. This requires advanced physiological images to help clinicians screen, and oxygen metabolism imaging is an important marker of physiological images [3].

Cerebral venous oxygen saturation (SvO2) is an important indicator to reflect the functional activities of brain tissue [4]. When brain tissue is in the state of hypoperfusion, the reduction of cerebral blood flow velocity leads to a decrease in local cerebral SvO2. Even if the blood flow recovers after treatment, the neural function will not recover without the improvement of cerebral SvO2 [5,6]. As we know, the change in the functional activity of brain tissue after stroke is the key to the prognosis of patients. Therefore, the measurement of cerebral SvO2 in patients with AIS is essential for assessing stroke severity, treatment, and prognosis.

Cerebral SvO2 can be measured using ^15^O_2_-positron emission tomography, which is considered the gold standard [7]. However, the spatial resolution of the positron emission tomography is low, and a radiolabeled isotope medium needs to be injected intravenously; thus, it is not routinely applied in clinical research. Methods based on magnetic resonance imaging (MRI) to measure cerebral SvO2 have been proposed by researchers [8,9,10,11,12,13]. Quantitative susceptibility mapping (QSM) can measure the whole brain or local venous oxygen saturation based on paramagnetic deoxyhemoglobin in venous vessels [14]. Compared with other imaging methods, QSM has the advantages of high resolution and high signal-to-noise ratio [15].

QSM has been used to explore cerebral SvO2 in healthy individuals, patients with traumatic brain injury, and a preclinical rat model of stroke [16,17,18,19,20]. Recently, many researchers have measured SvO2 in the asymmetrically prominent cortical veins (APCV) region of stroke patients based on QSM. Xia et al. [21] were the first to apply the measurement of cerebral SvO2 based on QSM to study AIS. They calculated that the cerebral SvO2 in the APCV region decreased by 16–44%, and these abnormal veins can be identified using a susceptibility threshold. Luo et al. [22] found that the National Institutes of Health Stroke Scale (NIHSS) scores increased in 31 patients with APCV on QSM at admission, indicating an unfavorite prognosis.

In the above studies, researchers all used SPIN software (nuclear magnetic resonance signal processing) to calculate local cerebral SvO2 values after manually drawing APCV regions. However, the manual drawing method depends more on the experience of clinicians and has an impact on the calculation results of cerebral SvO2. Due to the disadvantages of subjectivity, non-repeatability, and time requirements, this method cannot be applied in clinical research. In addition, SvO2 in the brain is usually described as an average value, which cannot directly show the changes in oxygen metabolism in brain tissue.

This study designed a set of automated measurement methods for use in patients with AIS. We can obtain not only the images of abnormal venous vessels, but also the quantitative distribution of SvO2 in the ischemic hemisphere based on the 1.5 Tesla MR equipment. With the help of the distribution, cerebral SvO2 in the hypoxic region was calculated to explore the guiding significance in predicting the early prognosis of patients with AIS.

## 2. Materials and Methods

### 2.1. Patients

This was a retrospective study of 537 patients with AIS admitted to the neurology department of our hospital between 2017 and 2019. The inclusion criteria for this study were: (1) Baseline MR examination (within 24 h after symptom onset) and the follow-up (at discharge); (2) Complete sequences: diffusion-weighted imaging, perfusion-weighted imaging, magnetic resonance angiography, and susceptibility-weighted imaging (SWI); (3) Occlusions of the M1 segment of the middle cerebral artery; (4) APCV coverage was a majority of the ipsilateral hemisphere, and consistent with increased delay times seen on mean transit time; (5) NIHSS scores (0–25) (baseline and follow-up) and 90-day modified Rankin Scale (mRS) scores (0–6). Exclusion criteria: (1) chronic lesions of cerebral infarction; (2) brain injury, brain tumor, and other neurological diseases; (3) SWI image artifacts were serious. Finally, 39 cases were included in the study.

### 2.2. MR Examinations

All MRI data were collected on a 1.5-Tesla Avanto scanner (Siemens, Erlangen, Germany). The scanning parameters: SWI: slices, 72; slice thickness, 1.6 mm; pixel spacing, 0.718 × 0.718 mm^2^; repetition time, 79 ms; echo time, 40 ms; bandwidth, 80 Hz/pixel; field of view, 230 × 230 mm^2^; and matrix size, 260 × 320. Perfusion-weighted imaging: slices, 19; slice thickness, 5 mm; pixel spacing, 0.89 × 0.89 mm^2^; repetition time, 1520 ms; echo time, 32 ms; bandwidth, 1346 Hz/pixel; field of view, 230 × 230 mm^2^; matrix size, 128 × 128; and measurements, 50. Diffusion-weighted imaging: slices, 18; slice thickness, 5 mm; pixel spacing, 1.198 × 1.198 mm^2^; repetition time, 3600 ms; echo time, 102 ms; bandwidth, 964 Hz/pixel; field of view, 230 × 230 mm^2^; matrix size, 192 × 192; b = 1000 s/mm^2^; and EPI factor: 192. Gadopentetate dimeglumine (Shanghai Pharmaceutical Corporation, Shanghai, China) with a dose of 0.2 mmol/kg body weight and a saline flush of 30 mL was injected at a flow rate of 4 mL/s.

### 2.3. Quantitative Distribution of SvO2

According to the relationship between susceptibility and cerebral SvO2, we used QSM images to measure local cerebral SvO2. Firstly, the QSM was automatically reconstructed using the amplitude and phase images obtained at admission or discharge. Then, the cerebral SvO2 of each ROI in the ischemic hemisphere was calculated. Finally, the cerebral SvO2 distribution map with the QSM images was displayed together.

#### 2.3.1. Theory of Measuring SvO2

The susceptibility difference ($X_{vein-tissue}$) between veins ($X_{vein}$) and surrounding no-blood tissues $\left(X_{tissue}\right)$ is directly correlated with SvO2 based on the following equation [23]:

(1) $$\Delta X_{vein-tissue}=K\cdot \Delta \chi_{do}\cdot \mathrm{Hct}(1-\mathrm{SvO}2)\;$$

where *Κ* is a constant depending on geometry and the magnetic field strength. $\chi_{do}$ is the difference in susceptibility per unit hematocrit between totally oxygenated blood and deoxygenated blood (4π × 0.27 ppm) [24]. Hct stands for hematocrit fractional value in large draining veins (normal range: 37–47% for women and 39–50% for men) [25]. In this paper, we assume the susceptibility of non-blood tissue $X_{tissue}=0$ [19]. Therefore, Δ$X_{vein-tissue}$ was equal to $X_{vein}$. This study provides susceptibility in ppb (10^−9^).

Taking the ratio of the changes in oxygen saturation normalized to the initial values leads to the following simplified equation, where all other constants have been canceled out:

(2) $$\Delta \mathrm{SvO}2_{\left(ab\right)}=-\left(1-\mathrm{SvO}2_{\left(n\right)}\right)\cdot \Delta X_{ab}∕X_{n}\;$$

$\Delta X_{ab}=X_{n}-X_{ab}$ is the susceptibility difference between the ischemic ($X_{ab}$) and control hemispheres ($X_{n}$). For patients with AIS, $\mathrm{SvO}2_{\left(n\right)}$ is the oxygen saturation level of the control hemisphere, assuming that the value is 70% [26]. $\mathrm{SvO}2_{\left(ab\right)}$ in the ischemic areas can be expressed as

(3) $$\mathrm{SvO}2_{\left(ab\right)}=\mathrm{SvO}2_{\left(n\right)}-\mathrm{SvO}2_{\left(ab\right)\;}\;$$

#### 2.3.2. Reconstructed QSM

**Figure 1:**
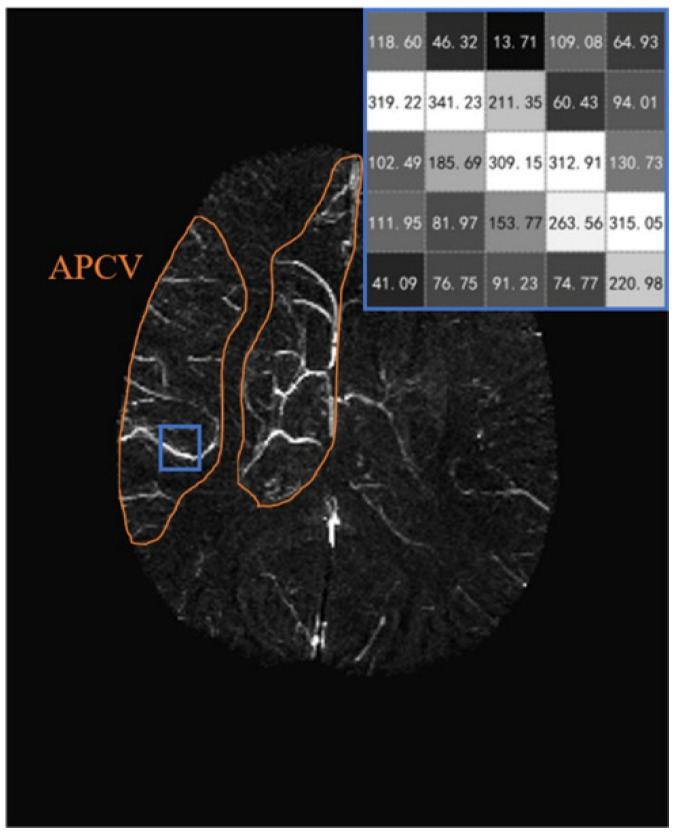
The susceptibility of venous vessels on quantitative susceptibility mapping-maximum intensity projection images (QSM-MIP) of a patient with acute ischemic stroke (AIS) with asymmetrically prominent cortical veins (APCV). APCV regions (orange region of interest) were drawn surrounding high-signal cortical veins (40/72 slices).

The reconstruction process for QSM was divided into three parts: initial processing, removal of background field, and susceptibility inversion. First, the Laplace method (Laplacian Operator ∇2) was used to complete the unwrapping of phase information through a fast Fourier transform [27]. Second, we adopted the most typical Sophisticated Harmonic Artifact Reduction for Phase Data method to remove the background field caused by the external susceptibility (such as air) based on the corresponding physical and mathematical properties [28]. Third, phase k-space was interpolated by zero filling the phase images to a larger matrix size to reduce aliasing artifacts—A regularization threshold of 0.1 was used for the inverse processing to generate the initial QSM image [29]. As the signal decays very fast, the signal-to-noise ratio in the veins was much lower than in the surrounding tissue. Therefore, the susceptibility value in veins can have extremely high or low values, which may lead to bias in the cerebral SvO2 distribution map. We eliminated extremely low values using MIPs, and the pixels with high values were eliminated by the empirical threshold (400 ppb). A maximum intensity projection image was generated over 16 slices of QSM data to display abnormal cortical veins (Figure 1).

#### 2.3.3. Generated Distribution Map

The mean transit time of the two hemispheres was separately calculated to determine the location of the focus of ischemic stroke (left or right). As the patients selected in this study only had an ischemic stroke in one hemisphere, we assumed that SvO2 in the control hemisphere had not changed [21]. When generating the distribution, only the SvO2 of the ROI units in the ischemic hemisphere were calculated.

When measuring the SvO2 of an ROI unit in the ischemic hemisphere, it was also necessary to calculate the susceptibility of the symmetrical ROI unit with the midline of the brain as the axis. For this, QSM images were first aligned and rotated so that they were balanced along the *Y*-axis. Then, left and right hemispheres were segmented to generate QSM-left and QSM-right images. After that, the mutual information method was used for QSM-left and QSM-right registration, in which the ischemic hemisphere was the reference sequence and the control hemisphere was the moving sequence. Finally, the QSM-left and QSM-right images were divided into grids of different sizes on each slice.

**Figure 2:**
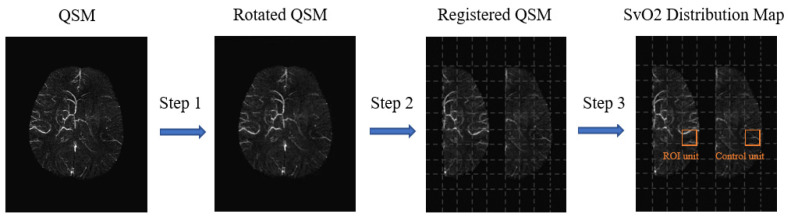
Schematic diagram of cerebral venous oxygen saturation (SvO2) distribution. Step 1: Rotated quantitative susceptibility map (QSM); Step 2: Registration of QSM-left and QSM-right; Step 3: Calculation of SvO2 of each region of interest (ROI) unit in the ischemic hemisphere.

The grid in the ischemic hemisphere was used as an ROI unit, and each unit had a symmetrical reference unit (control unit) in the control hemisphere. The mean value plus twice the standard deviation of the susceptibility of the veins in the control hemisphere was used to establish an upper threshold to extract abnormal veins. We calculated each ROI unit’s mean value of SvO2 according to Formulas (2) and (3). After measuring all units, the cerebral SvO2 distribution map was pseudo-colored and displayed jointly with QSM images (Figure 2).

#### 2.3.4. Parameters

For drawing a distribution map, it is very necessary to set the resolution, that is, to choose the specific ROI unit size. The resolution of our SWI data was 0.718 × 0.718 × 1.6 mm^3^. The diameter of cortical veins on the pia of healthy brains was about 1–2 mm, and the length of venous vessels was usually three or more times the diameter [30]. After a stroke, the susceptibility of the vein on the ischemic hemisphere may increase, and the diameter may become thicker on the QSM. To ensure that each ROI unit includes blood vessels and brain tissue, we set the minimum unit size to 3.59 × 3.59 × 1.6 mm^3^. At the same time, we also set two other unit sizes (7.18 × 7.18 × 1.6 mm^3^ and 14.36 × 14.36 × 1.6 mm^3^) to investigate the impact of different resolutions on the calculation results of cerebral SvO2.

**Figure 3:**
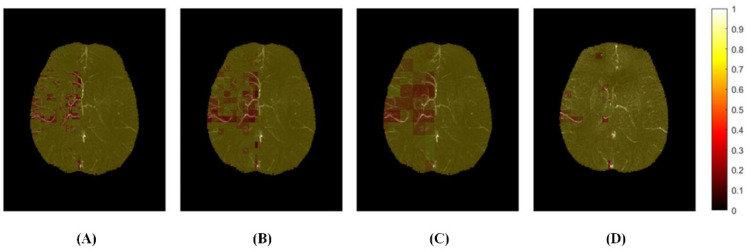
The distribution map of cerebral venous oxygen saturation (SvO2) with different sizes of regions of interest (ROI) of a patient (40/72 slices). (**A**) ROI size was 3.59 × 3.59 × 1.6 mm^3^; (**B**,**D**) ROI size was 7.18 × 7.18 × 1.6 mm^3^; (**C**) ROI size was 14.36 × 14.36 × 1.6 mm^3^; (**A**–**C**): At admission; (**D**): At discharge.

After calculating all ROI units in the ischemic hemisphere, the quantitative distribution of cerebral SvO2 was performed. ROI units with SvO2 value <0.7 were defined as hypoxic regions and were marked red on the oxygen saturation map. The darker the red, the more serious the hypoxia in this region was. The yellow ROIs indicate no change in oxygen metabolism in this region due to insufficient blood supply to brain tissue. We counted the number of pixels in hypoxic tissue and calculated the volume, which was defined as the hypoxic volume (Figure 3).

### 2.4. Evaluated

#### 2.4.1. Calculated Cerebral SvO2

The quantitative distribution map was used to automatically calculate the mean value of cerebral SvO2 in hypoxic regions and the volume of the hypoxic regions. The range of slices for the distribution was selected from the top layer to the middle layer of the brain, covering the cerebral cortical veins and eliminating the regions where iron deposits are most likely to occur [31]. In calculating cerebral SvO2 in APCV regions, we invited two radiologists with more than 7 years of experience in MRI diagnosis of the nervous system who manually drew the APCV on the QSM, and another radiologist with higher seniority who evaluated the drawn ROIs. For the controversial ROI, the three observers discussed and reached an agreement. Then, the mean value of cerebral SvO2 in APCV regions was calculated using SPIN software.

#### 2.4.2. Statistical Analysis

Statistical analyses were conducted using SPSS software (v. 26.0; IBM Corp., Armonk, NY, USA). Mean ± standard deviation was described for the continuous variables with normal distribution, and *n* (%) was described for categorical variables. The independent t-test or chi-square test was used to compare the statistical differences in clinical baseline factors between the favorable and unfavorable prognosis groups. The intraclass correlation coefficient (ICC) and Bland–Altman analysis were conducted to evaluate the agreement and bias of cerebral SvO2 in hypoxic with different resolution and APCV regions. An ICC > 0.75 was considered to represent an excellent agreement between measurements. The image resolution with the highest ICC value was defined as the best resolution, and the cerebral SvO2 distribution map with the best resolution will be used for the correlation analysis. Spearman correlation analyses were performed to analyze the relationship between MRI measurements and clinical scores (|r| > 0.3 and *p* < 0.05 was considered to indicate a significant correlation). Sample power analysis was conducted to evaluate the reliability of our statistical results (reliability: Power of test (1-β) more than 0.8 with significance level = 0.05). RAPID software was used to calculate the volume of infarct and hypoperfusion. Univariate and multivariate logistic regression analyses were conducted to calculate the odds ratios of cerebral SvO2 in predicting prognosis.

## 3. Results

### 3.1. Clinical Factors

When 90-mRS scores were 3–6, we considered that the patient had an unfavorable prognosis. Among all AIS patients (*n* = 39), 27 were in the favorable prognosis group and 12 in the unfavorable prognosis group. Table 1 summarized the clinical baseline factors of patients and the differences between the two groups. The percentage of patients with diabetes in the unfavorable prognosis group was significantly higher than that in the favorable prognosis group (66.7% vs. 25.9%). No significant differences in other baseline factors between the two groups (*p* > 0.05).

### 3.2. Comparison of SvO2 in Hypoxic Regions between Different Resolutions

**Figure 4:**
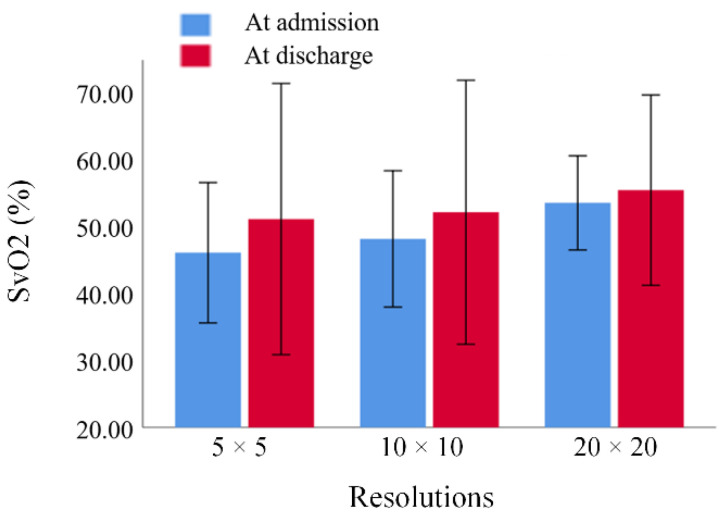
The cerebral venous oxygen saturation in hypoxic regions measured by different resolutions at admission and discharge of patients with acute ischemic stroke (5 × 5 ROI size: 3.59 × 3.59 × 1.6 mm^3^; 10 × 10 ROI size: 7.18 × 7.18 × 1.6 mm^3^; 20 × 20 ROI size: 14.36 × 14.36 × 1.6 mm^3^).

The mean value of cerebral SvO2 in hypoxic regions measured by the distribution map with 14.36 × 14.36 × 1.6 mm^3^ (admission: 53.97 ± 3.51% discharge: 55.89 ± 7.10%) was significantly greater than 3.59 × 3.59 × 1.6 mm^3^ (admission: 46.43 ± 5.24% discharge: 51.52 ± 10.12%) and 7.18 × 7.18 × 1.6 mm^3^ (admission: 48.52 ± 5.08% discharge: 52.54 ± 9.85%) (Figure 4).

### 3.3. Agreement on SvO2 between Different Regions

**Figure 5:**
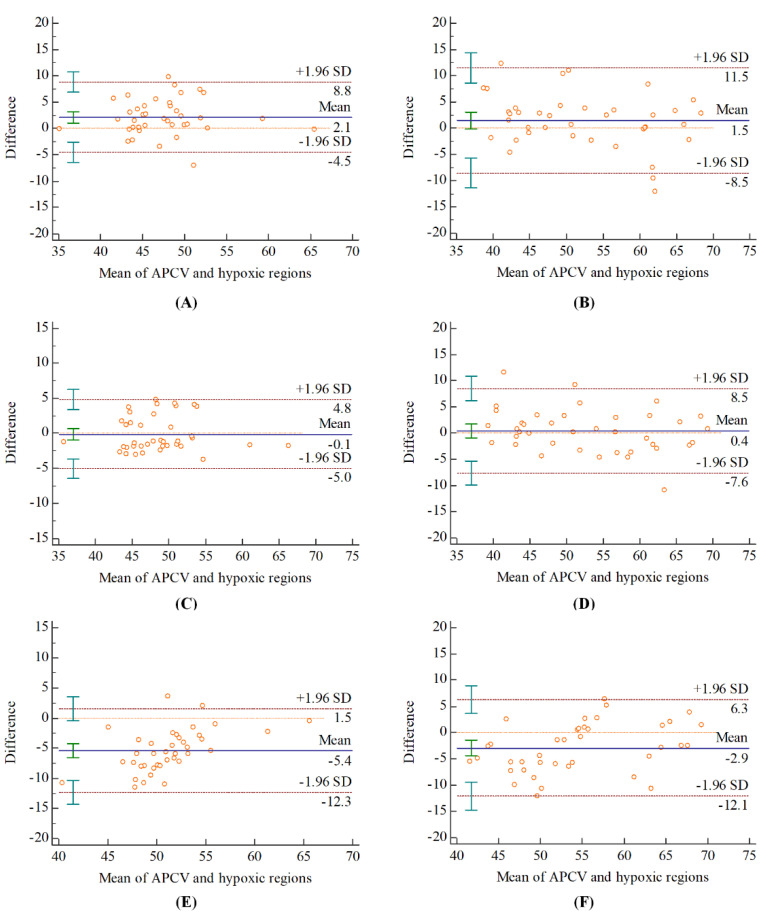
Bland−Altman analysis for the agreement in measuring the cerebral SvO2 between APCV regions and hypoxic regions measured by the quantitative distribution map with (**A**,**B**) 3.59 × 3.59 × 1.6 mm^3^; (**C**,**D**) 7.18 × 7.18 × 1.6 mm^3^; (**E**,**F**) 14.36 × 14.36 × 1.6 mm^3^ resolutions. (**A**,**C**,**E**) at admission; (**B**,**D**,**F**) at discharge.

The cerebral SvO2 values in APCV regions at admission and discharge were 48.57 ± 5.30% and 53.00 ± 8.78%, respectively. Table 2 summarized the ICC analysis results between cerebral SvO2 in hypoxic regions with different resolutions and APCV regions. The cerebral SvO2 in hypoxic regions and APCV regions were fair to a good agreement. Excellent agreement was found between the cerebral SvO2 in the hypoxic region with a resolution of 7.18 × 7.18 × 1.6 mm^3^ (best resolution) and the APCV region (ICC > 0.75). The Bland–Altman analysis showed that cerebral SvO2 in hypoxic regions measured by the distribution map with the best resolution has the smallest bias than other resolutions as compared with APCV regions (Figure 5).

### 3.4. Correlation between SvO2 and Clinical Scores

Spearman correlation analysis results showed that the cerebral SvO2 in hypoxic regions measured by distribution map with the best resolution was significantly negatively correlated with NIHSS scores (Table 3). The cerebral SvO2 and its changes measured at discharge were significantly negatively correlated with 90-day mRS scores. Similarly, NIHSS scores and infarct volume at discharge were also correlated with an early prognosis (Table 4). The volume of hypoxic regions was not significantly correlated with mRS scores (all |r| < 0.3). The difference between SvO2, NIHSS scores, and 90-day mRS score was significant at admission and discharge (all *p* < 0.01).

### 3.5. Association between SvO2 and Prognosis

Univariate and multivariate logistic regression analysis showed that cerebral SvO2 and its changes at discharge were significantly associated with unfavorable prognosis before and after adjusting baseline factors (age and gender) (*n* = 39, all power of test more than 0.8). Table 5 summarizes the results of the logistic regression analysis.

## 4. Discussion

The results demonstrated the validity and feasibility of the quantitative distribution map used to measure cerebral SvO2 in hypoxic areas in patients with AIS. Patients with AIS can be treated with intraarterial thrombolysis or thrombectomy within 4.5 h of onset, which is currently an active and effective treatment for acute cerebral infarction [32]. However, it usually takes an average of 15–20 min for each patient from QSM reconstruction to calculate cerebral SvO2 in APCV. Instead of manually marking the APCV regions on each slice by clinicians, the measurement method in this paper only took about 5 min, dramatically decreasing the time required to measure cerebral SvO2 in patients with AIS. In addition, it provided an objective and reproducible method to study the correlation between cerebral SvO2 in hypoxic regions and AIS development on large datasets.

The significant correlation between cerebral SvO2 in the hypoxic areas and NIHSS scores showed that it could be used to evaluate the clinical status of AIS. In previous studies, the changes of SvO2 in APCV correlated with the changes in NIHSS scores in patients with AIS (r = −0.37, *p* = 0.03) [33]. This was consistent with the correlation of cerebral SvO2 in hypoxic regions with NIHSS scores seen in our paper. The decreased SvO2 reflected lower activity in the brain tissue, with higher NIHSS scores at admission. When recanalization or collateral circulation was established, the cerebral SvO2 increased, and asymmetric cortical veins on QSM sequences may decrease in number or even disappear. Meanwhile, the patient’s discomfort symptoms improved, and the NIHSS score decreased at discharge.

Long-term hypoxia of brain tissue after stroke will lead to functional neural injury. The cerebral SvO2 and its changes after treatment in hypoxic regions measured by the distribution map were strongly associated with patient prognosis. They could be used as a new independent imaging indicator. In previous studies, the patient’s early prognosis was also correlated with NIHSS scores and infarct volume at discharge [34]. However, the NIHSS scores, a commonly used clinical indicator to evaluate neurological brain function, have a particular subjectivity and depend on clinician experience. The same patient may be given different scores from different physicians, so it has some disadvantages as an indicator to evaluate the clinical status and prognosis of AIS.

In previous studies, a larger infarct volume at discharge corresponded to a worse clinical prognosis [35]. However, some patients with large infarct volume had an excellent clinical outcome at follow-up. In contrast, some patients with low infarct volume did not achieve the desired outcomes, which might be related to the fact that the perfusion results reflect changes in blood flow in vessels rather than neural activity. Recently, it has also been shown that the reduction of infarct volume was only part of the effects of endovascular treatment. The prognosis also depended on the location of the infarct [36].

The volume of hypoxic regions presented no significant correlation with NIHSS and mRS scores. This may be related to a part of leptomeningeal collateral circulation formation after occlusion of the middle cerebral artery. In previous studies, the APCV region was greater when leptomeningeal collateral circulation was less or absent [37]. Conversely, the smaller the region, the more leptomeningeal collateral circulation occurred. Therefore, the volume of hypoxic regions calculated by distribution map with 7.18 × 7.18 × 1.6 mm^3^ resolution cannot be used as an independent parameter to evaluate AIS. However, it may become a reference indicator for assessing collateral circulation in further study.

This study had several limitations. The setting of the empirical threshold for eliminating extremely high values on QSM may affect the calculation results of cerebral SvO2. The quantitative distribution map proposed in this paper can only show the region where SvO2 decreases, but cannot show hyperoxia regions. This method only applied to AIS patients with APCV regions on QSM images. For patients without APCV regions, QSM imaging with higher field strength MRI equipment is required, which may detect changes in cerebral SvO2. The reconstructed QSM images and statistical results were analyzed based on single-echo sequence acquisition. Because of the lack of multi-echo data, the consistency of statistical results between single-echo and multi-echo sequences has not been verified. In addition, the data came from a single center, and the relatively small dataset may also lead to a deviation in statistical results.

## 5. Conclusions

This study has proposed a method for automatically generating a quantitative distribution map of cerebral SvO2 in patients with AIS. This map not only measured cerebral SvO2 in hypoxic regions but also directly visualized abnormal venous distribution after stroke. The automated data processing method made it possible to comprehensively analyze the correlation of cerebral SvO2 changes with AIS progression on large datasets. Correlation analysis with NIHSS and mRS scores showed that the cerebral SvO2 in hypoxic regions could serve as an essential imaging indicator for evaluating the clinical status and early prognosis of AIS.

## Figures and Tables

**Table 1:** The clinical baseline factors of all patients.

Factors	ALL (*n* = 39)	Favorable (*n* = 27)	Unfavorable (*n* = 12)	*p*
Age(years)	70.0 ± 10.5	68.9 ± 11.0	72.7 ± 9.1	0.299
Sex(male)	27 (69.2%)	20 (74.1%)	7 (58.3%)	0.326
Hypertension	30 (76.9%)	22 (81.5%)	8 (66.7%)	0.311
Diabetes	15 (38.5%)	7 (25.9%)	8 (66.7%)	0.016
Atrial fibrillation	11 (28.2%)	9 (33.3%)	2 (16.7%)	0.286
NIHSS ^1^	6.2 ± 5.7	5.8 ± 5.9	7.2 ± 5.5	0.492

^1^ NIHSS: National Institutes of Health Stroke Scale.

**Table 2:** Intraclass correlation coefficient analysis results in cerebral venous oxygen saturation in different regions.

Resolution (mm^3^)	At Admission	At Discharge
3.59 × 3.59 × 1.6	0.741, 95% CI ^1^: 0.435–0.875	0.852, 95% CI: 0.733–0.920
7.18 × 7.18 × 1.6	0.879, 95% CI: 0.782–0.935	0.906, 95% CI: 0.828–0.949
14.36 × 14.36 × 1.6	0.412, 95% CI: −0.103–0.740	0.784, 95% CI:0.509–0.898

^1^ CI: Confidence Interval.

**Table 3:** Correlation between cerebral venous oxygen saturation and clinical scores (*n* = 39).

Clinical Scores	At Admission		At Discharge		Changes	
	Spearman	1-β ^4^	Spearman	1-β	Spearman	1-β
NIHSS (1) ^1^	−0.452 **	0.842	0.140	0.136	0.335	0.562
NIHSS (2)	−0.246	0.330	−0.507 **	0.924	−0.353 *	0.611
ΔNIHSS ^2^	0.384	0.691	−0.531 **	0.945	−0.661 **	0.997
90-day mRS ^3^	−0.177	0.190	−0.619 **	0.992	−0.463 **	0.862

^1^ NIHSS: National Institutes of Health Stroke Scale; 1: measured at admission; 2: at discharge. ^2^ Δ: the difference between admission and discharge. ^3^ mRS: modified Rankin Scale. ^4^ 1-β: Power of test. *: *p* < 0.05, significant correlation; **: *p* < 0.01, significant correlation.

**Table 4:** Risk factors for 90-day modified Rankin scale scores (*n* = 39).

Parameter		Spearman	1-β ^3^
90-day mRS	NIHSS (1) ^1^	0.217	0.266
	NIHSS (2)	0.770 **	0.999
	ΔNIHSS ^2^	−0.379 *	0.679
	MRI measurement		
	Infarct volume (1)	0.149	0.148
	Infarct volume (2)	0.547 **	0.962
	Δ Infarct volume	−0.525 **	0.943
	Hypoperfusion volume (1)	−0.108	0.100
	Hypoperfusion volume (2)	0.284	0.425
	Δ Hypoperfusion volume	−0.012	0.051
	Hypoxia volume (1)	0.121	0.113
	Hypoxia volume (2)	0.125	0.118
	Δ Hypoxia volume	−0.023	0.052

^1^ NIHSS: National Institutes of Health Stroke Scale; 1: measured at admission; 2: at discharge. ^2^ Δ: the difference between admission and discharge. ^3^ 1-β: Power of test. *: *p* < 0.05, significant correlation; **: *p* < 0.01, significant correlation.

**Table 5:** Association between cerebral venous oxygen saturation and unfavorable prognosis.

Indicators	Univariate Logistic Regression			Multivariate Logistic Regression		
	OR ^3^	95% CI ^4^	*p*	OR	95% CI	*p*
SvO2 (1) ^1^	0.951	0.822–1.101	0.500	0.950	0.818–1.104	0.504
SvO2 (2) ^1^	0.849	0.757–0.952	0.005	0.812	0.701–0.941	0.006
ΔSvO2 ^2^	0.902	0.831–0.980	0.015	0.886	0.804–0.975	0.013

^1^ SvO2: venous oxygen saturation; 1: measured at admission; 2: measured at discharge. ^2^ Δ: the difference between admission and discharge. ^3^ OR: Odds Ratio. ^4^ CI: Confidence Interval.

## Data Availability

The data presented in this study are available on request from the corresponding author. The data are not publicly available due to ethical restrictions.
